# Tetra­aqua­bis­{3-carb­oxy-5-[(4-carb­oxy­phen­yl)diazen­yl]benzoato-κ*O* ^1^}cobalt(II) dihydrate

**DOI:** 10.1107/S1600536811045557

**Published:** 2011-11-05

**Authors:** Liang Bai, Jun Zhao

**Affiliations:** a College of Mechanical & Material Engineering, China Three Gorges University, Yichang 443002, People’s Republic of China

## Abstract

In the title complex, [Co(C_15_H_9_N_2_O_6_)_2_(H_2_O)_4_]·2H_2_O, the Co^II^ ion is located on an inversion center and is coordinated by two monodentate 3-carb­oxy-5-[(4-carb­oxy­phen­yl)diazen­yl]benzo­ate ligands and four water mol­ecules in a distorted octa­hedral geometry. In the crystal, inter­molecular O—H⋯O hydrogen bonds link the mol­ecules into a three-dimensional supra­molecular network.

## Related literature

For background to coordination polymers, see: Kitagawa *et al.* (2004 ▶); Moulton & Zaworotko (2001 ▶).
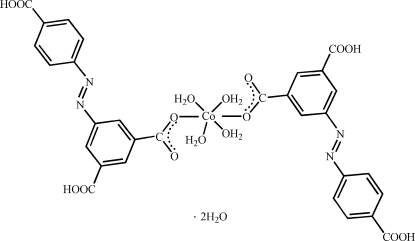


## Experimental

### 

#### Crystal data

[Co(C_15_H_9_N_2_O_6_)_2_(H_2_O)_4_]·2H_2_O
*M* *_r_* = 793.51
Monoclinic, 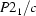

*a* = 19.347 (10) Å
*b* = 7.105 (3) Å
*c* = 12.379 (6) Å
β = 103.020 (9)°
*V* = 1657.9 (14) Å^3^
*Z* = 2
Mo *K*α radiation
μ = 0.61 mm^−1^
*T* = 296 K
0.26 × 0.21 × 0.18 mm

#### Data collection

Bruker APEX CCD diffractometer
Absorption correction: multi-scan (*SADABS*; Sheldrick, 1996 ▶) *T*_min_ = 0.858, *T*_max_ = 0.896
16990 measured reflections
3795 independent reflections
3435 reflections with *I* > 2σ(*I*)
*R*_int_ = 0.055

#### Refinement

*R*[*F* ^2^ > 2σ(*F* ^2^)] = 0.038
*wR*(*F* ^2^) = 0.105
*S* = 1.02
3795 reflections
253 parameters
9 restraints
H atoms treated by a mixture of independent and constrained refinement
Δρ_max_ = 0.29 e Å^−3^
Δρ_min_ = −0.47 e Å^−3^

### 

Data collection: *SMART* (Bruker, 2007 ▶); cell refinement: *SAINT* (Bruker, 2007 ▶); data reduction: *SAINT*; program(s) used to solve structure: *SHELXS97* (Sheldrick, 2008 ▶); program(s) used to refine structure: *SHELXL97* (Sheldrick, 2008 ▶); molecular graphics: *SHELXTL* (Sheldrick, 2008 ▶); software used to prepare material for publication: *SHELXTL*.

## Supplementary Material

Crystal structure: contains datablock(s) I, global. DOI: 10.1107/S1600536811045557/hy2481sup1.cif

Structure factors: contains datablock(s) I. DOI: 10.1107/S1600536811045557/hy2481Isup2.hkl

Additional supplementary materials:  crystallographic information; 3D view; checkCIF report

## Figures and Tables

**Table 1:** Hydrogen-bond geometry (Å, °)

*D*—H⋯*A*	*D*—H	H⋯*A*	*D*⋯*A*	*D*—H⋯*A*
O4—H4*A*⋯O5^i^	0.82	1.82	2.605 (2)	160
O6—H6*A*⋯O1^ii^	0.82	1.78	2.574 (2)	164
O7—H7*A*⋯O3^iii^	0.83 (1)	1.92 (1)	2.746 (2)	170 (3)
O7—H7*B*⋯O1*W*^iv^	0.82	2.05	2.791 (2)	151
O8—H8*A*⋯O1	0.82	1.98	2.697 (2)	145
O8—H8*B*⋯O1*W*	0.87 (1)	1.94 (1)	2.797 (2)	169 (2)
O1*W*—H1*WA*⋯O8^v^	0.85 (1)	2.12 (1)	2.957 (2)	169 (3)
O1*W*—H1*WB*⋯O3^vi^	0.85 (1)	2.15 (2)	2.937 (2)	155 (3)

Symmetry codes: (i) 
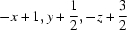
; (ii) 
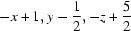
; (iii) 
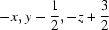
; (iv) 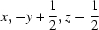; (v) 
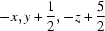
; (vi) 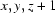.

**Fig. 1.**
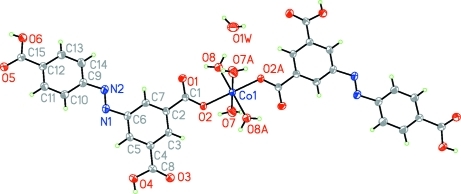
The molecular structure of the title compound, showing the 50% probability displacement ellipsoids. [Symmetry code: (A) -x, -y+1, -z+2.]

**Fig. 2.**
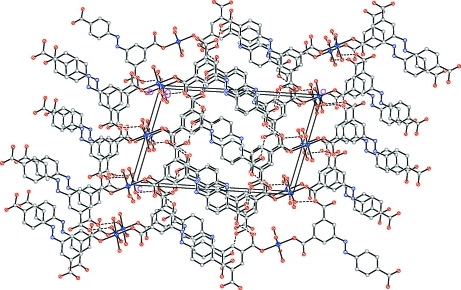
The crystal packing of the title compound, showing the three-dimensional network structure formed by hydrogen bonding interactions (dashed lines). H atoms are omitted for clarity.

## supplementary crystallographic information

### Comment

The formation of coordination polymers is an active area of research as these compounds have potential uses in gas storage, molecular sieves, magnetism and so on (Kitagawa *et al.*, 2004; Moulton & Zaworotko, 2001). During the synthesis of polymeric complexes using 5-[(4-carboxyphenyl)diazenyl]isophthalate (*L*) as bridging ligand, to our surprise, the title monomeric Co(II) complex was obtained.

The title complex is a centrosymmetric mononuclear complex. The Co^II^ ion, which is located on an inversion center, is six-coordinated by two carboxylate O atoms from two *L* ligands and four water O atoms, resulting in a distorted octahedral geometry (Fig. 1). In the *L* ligand, two benzene rings is almost coplanar and the dihedral angle is 4.62 (4)°. A three-dimensional supramolecular network structure is formed through the extended hydrogen bonding interactions between water molecules and carboxylate O atoms (Table 1, Fig. 2).

### Experimental

A mixture of 5-[(4-carboxyphenyl)diazenyl]isophthalic acid (0.031 g, 0.1 mmol), Co(CH_3_CO_2_)_2_.4H_2_O (0.025 g, 0.1 mmol) and water (10 ml) was stired vigorously for 30 min and then sealed in a Teflon-lined stainless-steel autoclave. The autoclave was heated and maintained at 393 K for 3 days and then cooled to room temperature at 5 K h^-1^. Red prism crystals suitable for X-ray analysis were obtained.

### Refinement

H atoms of water molecules were identified from a difference Fourier map and refined with a restraint of O—H = 0.85 (1) Å and with *U*_iso_(H) = 1.5*U*_eq_(O). The other H atoms were positioned geometrically and refined using a riding model, with C—H = 0.93, O—H = 0.82 Å and *U*_iso_(H) = 1.2*U*_eq_(C) or 1.5*U*_eq_(O).

### Crystal data

**Table:**

[Co(C_15_H_9_N_2_O_6_)_2_(H_2_O)_4_]·2H_2_O	*F*(000) = 818
*M**_r_* = 793.51	2
Monoclinic, *P*2_1_/*c*	*D*_x_ = 1.590 Mg m^−^^3^
Hall symbol: -P 2ybc	Mo *K*α radiation, λ = 0.71073 Å
*a* = 19.347 (10) Å	Cell parameters from 4430 reflections
*b* = 7.105 (3) Å	θ = 3.1–27.5°
*c* = 12.379 (6) Å	µ = 0.61 mm^−^^1^
β = 103.020 (9)°	*T* = 296 K
*V* = 1657.9 (14) Å^3^	Prism, red
*Z* = 2	0.26 × 0.21 × 0.18 mm

### Data collection

**Table:**

Bruker APEX CCD diffractometer	3795 independent reflections
Radiation source: fine-focus sealed tube	3435 reflections with *I* > 2σ(*I*)
graphite	*R*_int_ = 0.055
φ and ω scans	θ_max_ = 27.5°, θ_min_ = 3.1°
Absorption correction: multi-scan (*SADABS*; Sheldrick, 1996)	*h* = −25→25
*T*_min_ = 0.858, *T*_max_ = 0.896	*k* = −9→9
16990 measured reflections	*l* = −16→16

### Refinement

**Table:**

Refinement on *F*^2^	Primary atom site location: structure-invariant direct methods
Least-squares matrix: full	Secondary atom site location: difference Fourier map
*R*[*F*^2^ > 2σ(*F*^2^)] = 0.038	Hydrogen site location: inferred from neighbouring sites
*wR*(*F*^2^) = 0.105	H atoms treated by a mixture of independent and constrained refinement
*S* = 1.02	*w* = 1/[σ^2^(*F*_o_^2^) + (0.0577*P*)^2^ + 0.5749*P*] where *P* = (*F*_o_^2^ + 2*F*_c_^2^)/3
3795 reflections	(Δ/σ)_max_ < 0.001
253 parameters	Δρ_max_ = 0.29 e Å^−^^3^
9 restraints	Δρ_min_ = −0.47 e Å^−^^3^

### Fractional atomic coordinates and isotropic or equivalent isotropic displacement parameters (Å^2^)

**Table:**

	*x*	*y*	*z*	*U*_iso_*/*U*_eq_	
Co1	0.0000	0.5000	1.0000	0.02445 (11)	
O5	0.74586 (6)	0.0750 (2)	1.15653 (10)	0.0380 (3)	
O6	0.71026 (7)	0.0661 (2)	1.31469 (10)	0.0449 (4)	
H6A	0.7499	0.0212	1.3377	0.067*	
O1	0.17152 (6)	0.4276 (2)	1.07663 (10)	0.0379 (3)	
N1	0.39438 (7)	0.3211 (2)	0.92114 (12)	0.0314 (3)	
N2	0.41162 (7)	0.2937 (2)	1.02335 (12)	0.0345 (3)	
O8	0.05213 (6)	0.35528 (19)	1.14721 (10)	0.0334 (3)	
H8A	0.0949	0.3524	1.1506	0.050*	
C7	0.27274 (8)	0.3904 (2)	0.94775 (13)	0.0263 (3)	
H7C	0.2866	0.3648	1.0232	0.032*	
O3	0.15235 (7)	0.5636 (3)	0.55330 (11)	0.0453 (4)	
C2	0.20334 (8)	0.4436 (2)	0.90172 (13)	0.0250 (3)	
O7	−0.03319 (7)	0.2493 (2)	0.91850 (12)	0.0435 (3)	
H7B	0.0013	0.1921	0.9072	0.065*	
C12	0.62389 (8)	0.1432 (2)	1.15653 (13)	0.0278 (3)	
O2	0.08675 (7)	0.48577 (19)	0.92636 (10)	0.0340 (3)	
C5	0.30177 (8)	0.4156 (3)	0.76885 (13)	0.0283 (3)	
H5A	0.3347	0.4056	0.7247	0.034*	
C1	0.15001 (8)	0.4543 (2)	0.97381 (13)	0.0258 (3)	
C6	0.32179 (8)	0.3752 (2)	0.88120 (13)	0.0268 (3)	
O4	0.26546 (7)	0.5081 (2)	0.55360 (11)	0.0396 (3)	
H4A	0.2520	0.5338	0.4876	0.059*	
C9	0.48438 (9)	0.2430 (3)	1.06321 (14)	0.0311 (4)	
C15	0.69910 (9)	0.0914 (3)	1.20755 (13)	0.0289 (3)	
C10	0.53469 (9)	0.2432 (3)	0.99908 (14)	0.0338 (4)	
H10A	0.5216	0.2765	0.9246	0.041*	
C3	0.18298 (9)	0.4847 (2)	0.78833 (14)	0.0265 (3)	
H3A	0.1366	0.5210	0.7572	0.032*	
C11	0.60397 (9)	0.1942 (3)	1.04523 (14)	0.0339 (4)	
H11A	0.6375	0.1953	1.0019	0.041*	
C14	0.50395 (10)	0.1931 (4)	1.17404 (16)	0.0461 (5)	
H14A	0.4705	0.1940	1.2175	0.055*	
C4	0.23232 (9)	0.4712 (2)	0.72194 (14)	0.0263 (3)	
C13	0.57344 (10)	0.1416 (3)	1.22021 (15)	0.0429 (5)	
H13A	0.5863	0.1057	1.2943	0.051*	
C8	0.21179 (9)	0.5190 (2)	0.60178 (14)	0.0287 (4)	
O1W	0.04442 (9)	0.5355 (2)	1.34499 (12)	0.0469 (4)	
H8B	0.0450 (9)	0.420 (4)	1.2030 (16)	0.070*	
H7A	−0.0701 (6)	0.190 (3)	0.919 (2)	0.070*	
H1WB	0.0831 (8)	0.557 (4)	1.3910 (19)	0.070*	
H1WA	0.0176 (11)	0.631 (3)	1.339 (2)	0.070*	

### Atomic displacement parameters (Å^2^)

**Table:**

	*U*^11^	*U*^22^	*U*^33^	*U*^12^	*U*^13^	*U*^23^
Co1	0.01671 (17)	0.0366 (2)	0.02086 (17)	−0.00116 (11)	0.00589 (12)	−0.00045 (11)
O5	0.0253 (6)	0.0628 (9)	0.0262 (6)	0.0058 (6)	0.0061 (5)	−0.0019 (6)
O6	0.0285 (7)	0.0810 (10)	0.0242 (6)	0.0165 (7)	0.0040 (5)	0.0094 (6)
O1	0.0218 (6)	0.0720 (9)	0.0200 (6)	−0.0032 (6)	0.0049 (4)	0.0026 (6)
N1	0.0205 (7)	0.0448 (8)	0.0285 (7)	0.0055 (6)	0.0048 (5)	0.0014 (6)
N2	0.0219 (7)	0.0525 (9)	0.0280 (7)	0.0057 (7)	0.0033 (5)	0.0038 (6)
O8	0.0256 (6)	0.0478 (7)	0.0274 (6)	0.0015 (5)	0.0070 (5)	0.0016 (5)
C7	0.0215 (7)	0.0362 (8)	0.0205 (7)	−0.0001 (6)	0.0035 (6)	0.0003 (6)
O3	0.0310 (7)	0.0787 (10)	0.0258 (6)	0.0186 (7)	0.0057 (5)	0.0077 (7)
C2	0.0188 (7)	0.0340 (8)	0.0229 (7)	−0.0008 (6)	0.0064 (6)	−0.0016 (6)
O7	0.0344 (7)	0.0473 (8)	0.0529 (8)	−0.0135 (6)	0.0184 (6)	−0.0153 (6)
C12	0.0240 (8)	0.0352 (8)	0.0232 (8)	0.0031 (7)	0.0031 (6)	−0.0002 (6)
O2	0.0168 (6)	0.0630 (9)	0.0231 (6)	0.0034 (5)	0.0067 (5)	0.0014 (5)
C5	0.0210 (7)	0.0403 (9)	0.0256 (8)	0.0020 (7)	0.0092 (6)	−0.0011 (7)
C1	0.0173 (7)	0.0379 (8)	0.0229 (8)	−0.0027 (6)	0.0058 (6)	−0.0022 (6)
C6	0.0185 (7)	0.0347 (8)	0.0264 (8)	0.0021 (6)	0.0034 (6)	−0.0006 (6)
O4	0.0295 (7)	0.0689 (10)	0.0226 (6)	0.0052 (6)	0.0104 (5)	0.0056 (5)
C9	0.0214 (8)	0.0427 (10)	0.0284 (8)	0.0040 (7)	0.0036 (6)	0.0012 (7)
C15	0.0252 (8)	0.0374 (9)	0.0231 (7)	0.0013 (7)	0.0036 (6)	−0.0014 (6)
C10	0.0267 (8)	0.0503 (11)	0.0237 (8)	0.0057 (8)	0.0044 (6)	0.0070 (7)
C3	0.0173 (7)	0.0385 (9)	0.0234 (8)	0.0016 (6)	0.0038 (6)	−0.0005 (6)
C11	0.0252 (8)	0.0515 (11)	0.0257 (8)	0.0059 (8)	0.0074 (6)	0.0044 (7)
C14	0.0279 (9)	0.0834 (16)	0.0284 (9)	0.0098 (10)	0.0096 (7)	0.0080 (9)
C4	0.0216 (8)	0.0355 (8)	0.0219 (8)	0.0008 (6)	0.0054 (6)	−0.0003 (6)
C13	0.0289 (9)	0.0767 (15)	0.0226 (8)	0.0104 (9)	0.0050 (7)	0.0091 (9)
C8	0.0253 (8)	0.0393 (9)	0.0226 (8)	0.0028 (7)	0.0074 (6)	−0.0013 (6)
O1W	0.0532 (10)	0.0503 (8)	0.0350 (8)	0.0024 (7)	0.0051 (7)	−0.0050 (6)

### Geometric parameters (Å, °)

**Table:**

Co1—O7^i^	2.0766 (15)	C12—C13	1.386 (2)
Co1—O7	2.0766 (15)	C12—C11	1.392 (2)
Co1—O2	2.0850 (15)	C12—C15	1.496 (2)
Co1—O2^i^	2.0850 (15)	O2—C1	1.253 (2)
Co1—O8^i^	2.1371 (14)	C5—C6	1.387 (2)
Co1—O8	2.1371 (14)	C5—C4	1.396 (2)
O5—C15	1.220 (2)	C5—H5A	0.9300
O6—C15	1.307 (2)	O4—C8	1.311 (2)
O6—H6A	0.8200	O4—H4A	0.8200
O1—C1	1.261 (2)	C9—C10	1.388 (2)
N1—N2	1.249 (2)	C9—C14	1.385 (3)
N1—C6	1.432 (2)	C10—C11	1.378 (2)
N2—C9	1.429 (2)	C10—H10A	0.9300
O8—H8A	0.8200	C3—C4	1.396 (2)
O8—H8B	0.867 (9)	C3—H3A	0.9300
C7—C2	1.388 (2)	C11—H11A	0.9300
C7—C6	1.394 (2)	C14—C13	1.386 (3)
C7—H7C	0.9300	C14—H14A	0.9300
O3—C8	1.213 (2)	C4—C8	1.490 (2)
C2—C3	1.401 (2)	C13—H13A	0.9300
C2—C1	1.511 (2)	O1W—H1WB	0.845 (10)
O7—H7B	0.8200	O1W—H1WA	0.847 (10)
O7—H7A	0.831 (9)		
			
O7^i^—Co1—O7	180.0	C4—C5—H5A	120.0
O7^i^—Co1—O2	93.60 (6)	O2—C1—O1	124.44 (15)
O7—Co1—O2	86.40 (6)	O2—C1—C2	117.21 (15)
O7^i^—Co1—O2^i^	86.40 (6)	O1—C1—C2	118.33 (14)
O7—Co1—O2^i^	93.60 (6)	C5—C6—C7	120.21 (15)
O2—Co1—O2^i^	180.0	C5—C6—N1	115.60 (14)
O7^i^—Co1—O8^i^	92.09 (6)	C7—C6—N1	124.19 (15)
O7—Co1—O8^i^	87.91 (6)	C8—O4—H4A	109.5
O2—Co1—O8^i^	85.55 (6)	C10—C9—C14	119.78 (16)
O2^i^—Co1—O8^i^	94.45 (6)	C10—C9—N2	124.45 (15)
O7^i^—Co1—O8	87.91 (6)	C14—C9—N2	115.77 (15)
O7—Co1—O8	92.09 (6)	O5—C15—O6	122.68 (16)
O2—Co1—O8	94.45 (6)	O5—C15—C12	124.70 (15)
O2^i^—Co1—O8	85.55 (6)	O6—C15—C12	112.62 (14)
O8^i^—Co1—O8	180.00 (4)	C11—C10—C9	120.31 (16)
C15—O6—H6A	109.5	C11—C10—H10A	119.8
N2—N1—C6	114.14 (13)	C9—C10—H10A	119.8
N1—N2—C9	113.99 (14)	C4—C3—C2	120.03 (15)
Co1—O8—H8A	109.5	C4—C3—H3A	120.0
Co1—O8—H8B	107.1 (19)	C2—C3—H3A	120.0
H8A—O8—H8B	108.1	C10—C11—C12	120.16 (16)
C2—C7—C6	120.20 (15)	C10—C11—H11A	119.9
C2—C7—H7C	119.9	C12—C11—H11A	119.9
C6—C7—H7C	119.9	C9—C14—C13	119.95 (17)
C7—C2—C3	119.73 (14)	C9—C14—H14A	120.0
C7—C2—C1	119.85 (14)	C13—C14—H14A	120.0
C3—C2—C1	120.41 (14)	C5—C4—C3	119.77 (15)
Co1—O7—H7B	109.5	C5—C4—C8	119.68 (15)
Co1—O7—H7A	127.2 (15)	C3—C4—C8	120.55 (15)
H7B—O7—H7A	118.8	C12—C13—C14	120.34 (17)
C13—C12—C11	119.45 (15)	C12—C13—H13A	119.8
C13—C12—C15	120.04 (15)	C14—C13—H13A	119.8
C11—C12—C15	120.52 (15)	O3—C8—O4	123.37 (16)
C1—O2—Co1	126.97 (12)	O3—C8—C4	124.27 (16)
C6—C5—C4	120.06 (15)	O4—C8—C4	112.36 (15)
C6—C5—H5A	120.0	H1WB—O1W—H1WA	110.4 (16)
			
C6—N1—N2—C9	−178.97 (15)	C13—C12—C15—O6	−6.3 (3)
C6—C7—C2—C3	−0.9 (3)	C11—C12—C15—O6	173.41 (17)
C6—C7—C2—C1	178.26 (16)	C14—C9—C10—C11	−0.1 (3)
O7^i^—Co1—O2—C1	69.81 (16)	N2—C9—C10—C11	179.18 (19)
O7—Co1—O2—C1	−110.19 (16)	C7—C2—C3—C4	0.3 (3)
O8^i^—Co1—O2—C1	161.62 (16)	C1—C2—C3—C4	−178.89 (15)
O8—Co1—O2—C1	−18.38 (16)	C9—C10—C11—C12	0.4 (3)
Co1—O2—C1—O1	−0.7 (3)	C13—C12—C11—C10	0.2 (3)
Co1—O2—C1—C2	177.75 (11)	C15—C12—C11—C10	−179.49 (18)
C7—C2—C1—O2	−173.46 (16)	C10—C9—C14—C13	−0.7 (3)
C3—C2—C1—O2	5.7 (2)	N2—C9—C14—C13	180.0 (2)
C7—C2—C1—O1	5.1 (3)	C6—C5—C4—C3	−0.5 (3)
C3—C2—C1—O1	−175.70 (17)	C6—C5—C4—C8	178.47 (16)
C4—C5—C6—C7	−0.2 (3)	C2—C3—C4—C5	0.4 (3)
C4—C5—C6—N1	−179.43 (16)	C2—C3—C4—C8	−178.52 (15)
C2—C7—C6—C5	0.9 (3)	C11—C12—C13—C14	−1.0 (3)
C2—C7—C6—N1	−179.93 (16)	C15—C12—C13—C14	178.7 (2)
N2—N1—C6—C5	176.98 (16)	C9—C14—C13—C12	1.2 (4)
N2—N1—C6—C7	−2.3 (3)	C5—C4—C8—O3	177.86 (18)
N1—N2—C9—C10	6.9 (3)	C3—C4—C8—O3	−3.2 (3)
N1—N2—C9—C14	−173.76 (19)	C5—C4—C8—O4	−2.3 (2)
C13—C12—C15—O5	174.2 (2)	C3—C4—C8—O4	176.63 (16)
C11—C12—C15—O5	−6.1 (3)		

Symmetry codes: (i) −*x*, −*y*+1, −*z*+2.

### Hydrogen-bond geometry (Å, °)

**Table:**

*D*—H···*A*	*D*—H	H···*A*	*D*···*A*	*D*—H···*A*
O4—H4A···O5^ii^	0.82	1.82	2.605 (2)	160
O6—H6A···O1^iii^	0.82	1.78	2.574 (2)	164
O7—H7A···O3^iv^	0.83 (1)	1.92 (1)	2.746 (2)	170 (3)
O7—H7B···O1W^v^	0.82	2.05	2.791 (2)	151
O8—H8A···O1	0.82	1.98	2.697 (2)	145
O8—H8B···O1W	0.87 (1)	1.94 (1)	2.797 (2)	169 (2)
O1W—H1WA···O8^vi^	0.85 (1)	2.12 (1)	2.957 (2)	169 (3)
O1W—H1WB···O3^vii^	0.85 (1)	2.15 (2)	2.937 (2)	155 (3)

Symmetry codes: (ii) −*x*+1, *y*+1/2, −*z*+3/2; (iii) −*x*+1, *y*−1/2, −*z*+5/2; (iv) −*x*, *y*−1/2, −*z*+3/2; (v) *x*, −*y*+1/2, *z*−1/2; (vi) −*x*, *y*+1/2, −*z*+5/2; (vii) *x*, *y*, *z*+1.
